# A Genotype-Phenotype Study of Multiple Hereditary Exostoses in Forty-Three Patients

**DOI:** 10.3390/jcm11133703

**Published:** 2022-06-27

**Authors:** Sungmin Kim, Chang-Hyun Lee, Seok-Yong Choi, Myeong-Kyu Kim, Sung Taek Jung

**Affiliations:** 1 Department of Orthopedic Surgery, Chonnam National University Medical School and Hospital, 42 Jebong-ro, Dong-gu, Gwangju 61469, Korea; kimsum83@gmail.com (S.K.); chlee5819@daum.net (C.-H.L.); 2 Department of Biomedical Sciences, Chonnam National University Medical School, Hwasun 58128, Korea; 3 Department of Neurology, Chonnam National University Medical School and Hospital, 42 Jebong-ro, Dong-gu, Gwangju 61469, Korea

**Keywords:** multiple hereditary exostoses, EXT1, EXT2, mutation, genotype, phenotype

## Abstract

Multiple hereditary exostoses (MHE) is a rare autosomal dominant skeletal disorder with a variety of clinical manifestations. We aimed to evaluate the general clinical phenotypic severity of MHE using our own scoring system and analyzed the risk factors associated with severe clinical phenotypes. In this study, 43 patients from 30 families were analyzed. The mutations were identified by direct sequencing of polymerase chain reaction-amplified genomic DNA or by multiplex ligation-dependent probe amplification. According to a new scoring system devised by the authors, the severity of the phenotype was assessed as mild, moderate, or severe based on the deformity of each segment, number of exostoses, leg length discrepancy, and functional limitations. Of 43 patients from 30 families, 39 patients (90.7%) and 24 families (80%) presented with EXT1 or EXT2 mutations. Patients with EXT1 mutations had a significantly worse phenotype than that of patients with EXT2 mutations or without any detectable mutation. The mean clinical score of patients with an EXT1 mutation (5.76; range, 2.0–8.0; SD = 1.60) was higher than that of patients with an EXT2 mutation (4.06; range, 2.0–7.0; SD = 1.47) or of those without any detectable mutation (4.63; range, 3.0–6.0; SD = 1.44; *p* = 0.005). According to our classification system, more patients with EXT1 mutations had ‘severe disease’ than those with EXT2 mutations. Deformity scores were also higher in patients with EXT1 mutations (*p* = 0.018). In the multivariate analysis, the deformity score was found to be associated with the ‘severe’ class (*p* = 0.031). In conclusion, 90.7% of patients with MHE showed EXT mutations. Our scoring system showed reliable results. We suggest that the extent of deformity is an important factor in determining the phenotype of MHE and close monitoring for the development of severe disease is recommended in patients with high deformity scores.

## 1. Introduction

Multiple hereditary exostoses (MHE) is an autosomal dominant disease characterized by multiple benign cartilage-capped osteochondromas, primarily at the metaphyseal region of long bones. These osteochondromas are caused by an increase in chondrocyte proliferation in children and result in excessive bone growth at the metaphysis. Lesions are usually evident and tend to grow in number and size through childhood and adolescence, until the closure of growth plates during puberty [1]. Clinical presentation in patients with MHE is heterogenous owing to the variable number, size, and location of tumors. The disruption of growth plates may lead to skeletal deformities, short stature, leg length discrepancy (LLD), or functional limitations [2]. Other complications include pain or sensory or motor deficits caused by the pressure applied to adjacent tissues [3]. In up to 5% of cases, osteochondromas can transform into malignant tumors, such as chondrosarcomas or osteosarcomas [4].

In almost 90% of patients with MHE, mutations are found in the genes coding for exostosin-1 (EXT1) and exostosin-2 (EXT2) proteins. EXT1 and EXT2 are ubiquitously expressed tumor-suppressor genes from the EXT family [5,6,7,8,9]. The EXT genes code for homologous Golgi-associated glycosyltransferases involved in the chain elongation step of heparan sulfate (HS) biosynthesis [7,8,9]. EXT1 and EXT2 form a heterooligomeric protein complex in the endoplasmic reticulum, which transfers to the Golgi apparatus where it catalyzes the chain elongation of HS chains, with the addition of N-acetylglucosamine and glucuronic acid [5,10,11]. HS plays an important role in the diffusion of Ihh, which regulates chondrocyte proliferation and differentiation [12,13]. Mutations in EXT1 or EXT2 are responsible for the premature termination and consequent loss of function of EXT proteins [14]. 

Several studies have reported on the genotype-phenotype correlation in MHEs [15,16,17,18,19]. Research has shown that EXT1 mutations are associated with disease severity [15,17,18] and malignant transformation [15]. In this study, we describe the clinical features of patients with MHE according to genotype and analyze the severity of their phenotype using our scoring system and investigate the risk factors associated with severe clinical phenotypes.

## 2. Materials and Methods

### 2.1. Patients

The study was approved by our Institutional Review Board (IRB ####-2020-280). We investigated 43 patients from 30 families at a single institution and obtained informed consent from all patients. A clinical diagnosis of MHE was made after obtaining an accurate medical history and performing a physical examination of the patient, including an evaluation of all palpable lesions, height, long bone deformities, and functional limitations. The genomic DNA of all patients was extracted from peripheral blood following standard protocol, and family histories were obtained.

### 2.2. Clinical Phenotype Study and Classification System

A new scoring system, “De-Nu-L-F score” was adopted in this study. The scoring system, which is based on the deformity of each segment, number of exostoses, LLD, and functional limitations, is delineated in Table 1. The total score is 10 (deformity, 4; functional limitation, 2; LLD, 2; and number of exostoses, 2); a higher total score indicates more severe clinical manifestation. A score of <4, 4 to 6, and >6 was graded as mild, moderate, and severe, respectively. 

Receiver operating characteristic (ROC) curves were created to assess the discrimination between EXT1 and EXT2, or EXT1 and no mutation, within our scoring system.

The radiological assessment consisted of a bilateral, upper-extremity anteroposterior view with the elbow extended and forearm supinated, and long-standing anteroposterior radiographs of the lower extremities, including the pelvis. To assess short bone deformity or involvement, we obtained radiographs of the feet and hands and anteroposterior and lateral views of the elbows, forearms, knees, and ankles in all patients. On these plain films, deformity, number of exostoses, and LLD were assessed.

**Figure 1:**
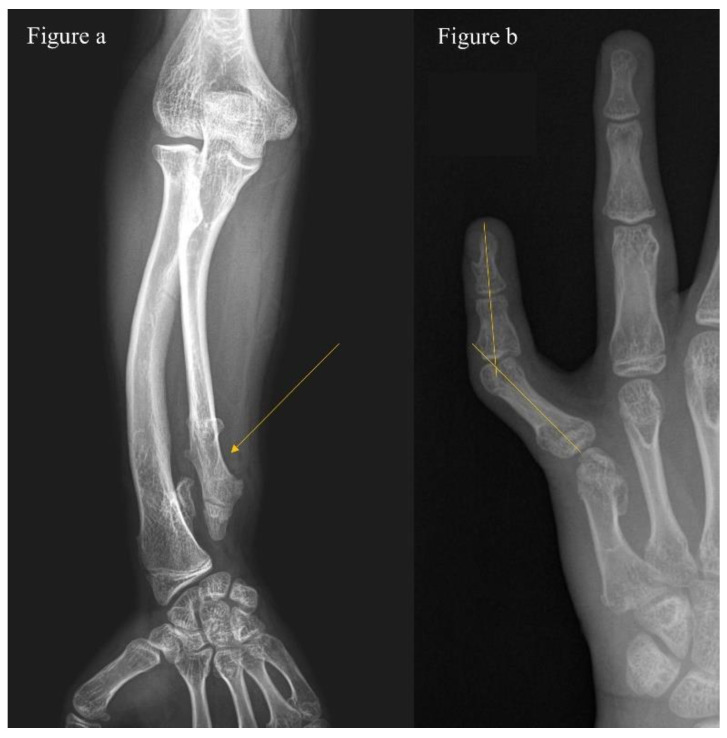
(**a**) A 12-year-old boy with multiple hereditary exostoses shows ulnar shortening and (**b**) radial angulation of the 5th finger due to an abnormal proximal phalanx.

**Figure 2:**
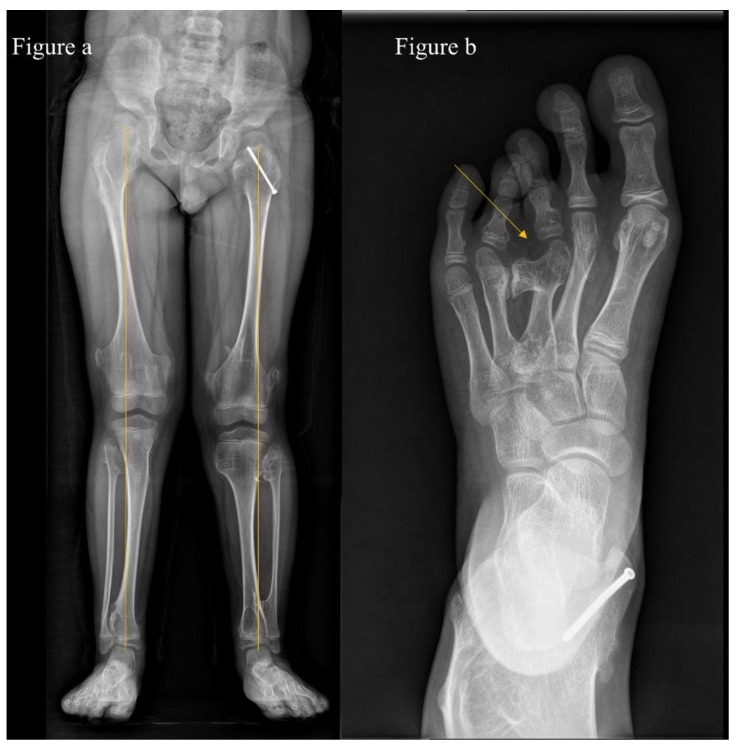
(**a**) An 8-year-old boy with multiple hereditary exostoses shows bilateral knee valgus deformity (14.2° on the left and 13.5° on the right) on the full-length standing anteroposterior view of hip-to-ankle and (**b**) foot deformity due to bony mass of the 3rd metatarsal head.

The assessment and scoring of deformities on each segment are shown in Table 1. A deformity of the upper extremity was considered when (1) there was more than 10 degrees of varus or valgus on the anteroposterior view of each segment (i.e., on the anteroposterior view of the forearm), or (2) there was shortening of the ulna or radius (Figure 1a). Where the elbow was dislocated or involvement was on both sides, we assigned an additional score. We assessed deformity of the hands and feet and considered it significant when we observed (1) more than 5 degrees of varus or valgus or (2) shortening (Figure 1b and Figure 2b). A deformity of lower extremities was considered when (1) there was more than 10 degrees of varus or valgus in an anteroposterior view of each segment (i.e., on the full-length standing anteroposterior view of hip-to-ankle, Figure 2a).

Patients were also evaluated for the number of exostoses, with individuals divided into three groups: those with fewer than 10 sites, those with 10 to 20 sites, and those with more than 20 sites. LLD was determined using full-length, standing, and anteroposterior hip-to-ankle radiographs. Leg length was measured from the center of the femoral head to the superior border of the talus. The LLDs were scored based on their severity (Table 1). We defined functional limitations using the criteria from the existing literature: (1) joint motion restricted by deformities or (2) functional impairment caused by the presence of exostoses. In addition, ‘pain’ was considered a functional limitation.

We also evaluated the heights of the patients. For each patient, stature percentile was determined according to national growth charts.

### 2.3. Molecular Screening

Genomic DNA mutation screening of EXT1 and EXT2 was performed for each patient. Genomic DNA was extracted from the peripheral blood of participants, and EXT1 and EXT2 were amplified by polymerase chain reaction (PCR). PCR products were run on an ABI 3730XL DNA Analyzer capillary sequencer (Applied Biosystems, Waltham, MA, USA, Forster City, CA, USA). For PCR negative patients, multiplex ligation-dependent probe amplification was performed using the SALSA MLPA probe mix P215-B4 EXT kit (MRC Holland, Amsterdam, The Netherlands) for the detection of deletions or duplications in the EXT1 and EXT2 genes. Data analysis and interpretation were performed using GeneMarker v3.0.1 software (Softgenetics, State College, PA, USA).

### 2.4. Statistics

Statistical analyses were performed using SPSS version 25.0 (SPSS, Chicago, IL, USA), with significance defined as *p* < 0.05. Data were assessed for normality on plots and with the Shapiro–Wilk test. Comparisons among the three groups were performed using one-way analysis of variance. Post hoc inferential analysis (Bonferroni’s test) was used to identify specific groups where significant differential expression occurred. The Pearson chi-square test was performed to investigate the relationships between grouping variables.

## 3. Results

**Figure 3:**
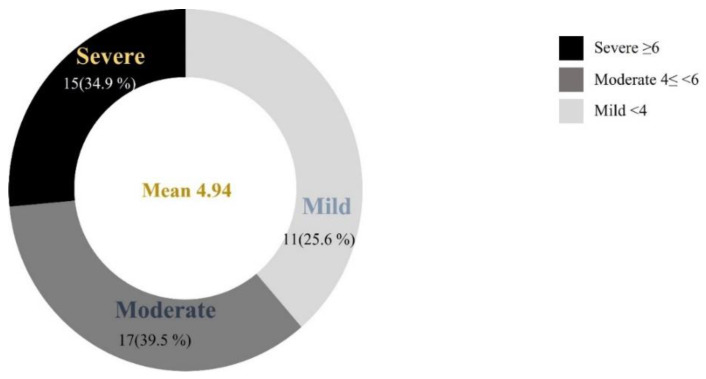
Distribution of patients according to the severity of phenotype based on our new scoring system (DeNuLF score).

Of 43 patients, belonging to 30 families, 39 patients (90.7%) and 24 families (80%) presented EXT1 or EXT2 mutations, 21 (48.8%) presented mutations in the EXT1 gene, and 18 (41.9%) in the EXT2 gene. Four patients (9.3%) had no identifiable mutations. The mean age was 24 years (range, 5–59), and the mean number of exostoses was 24.7 (range 7–52). The distribution of patients according to our scoring system is described in Figure 3. Area-under-the-curve (AUC) was 0.78 (95% confidence interval 0.638–0.928) and 0.79 (95% confidence interval 0.616–0.956).

Patients with an EXT1 mutation had a significantly worse phenotype than of those with an EXT2 mutation or of those without any detectable mutation (Table 2). The mean clinical score of patients with an EXT1 mutation (5.76; range, 2.0–8.0; SD, 1.60) was higher than that of patients with an EXT2 mutation (4.06; range, 2.0–7.0; SD, 1.47) or of those without any detectable mutation (4.63; range, 3.0–6.0; SD, 1.44; *p* = 0.005).

**Figure 4:**
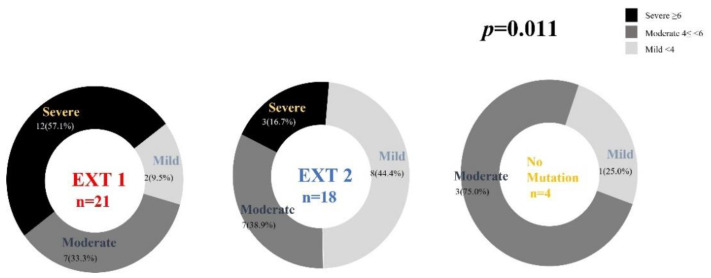
Phenotype–genotype correlations in patient with multiple hereditary exostoses according to the type of gene mutation.

Consequently, the proportion of patients with EXT1 mutations suffering from ‘severe’ disease was significantly higher than that of those with EXT2 mutations (*p* = 0.011, Figure 4). 

On the contrary, patients with EXT2 mutations tended to have milder disease. Deformity score was also higher in patients with EXT1 instead of EXT2 mutations (*p* = 0.018); however, there was no significant difference in familial history or sex according to the EXT1/EXT2 mutation (*p* = 0.066 and *p* = 0.814, respectively). Gene mutations in patients with MHE in our cohort are described in Table 3.

**Figure 5:**
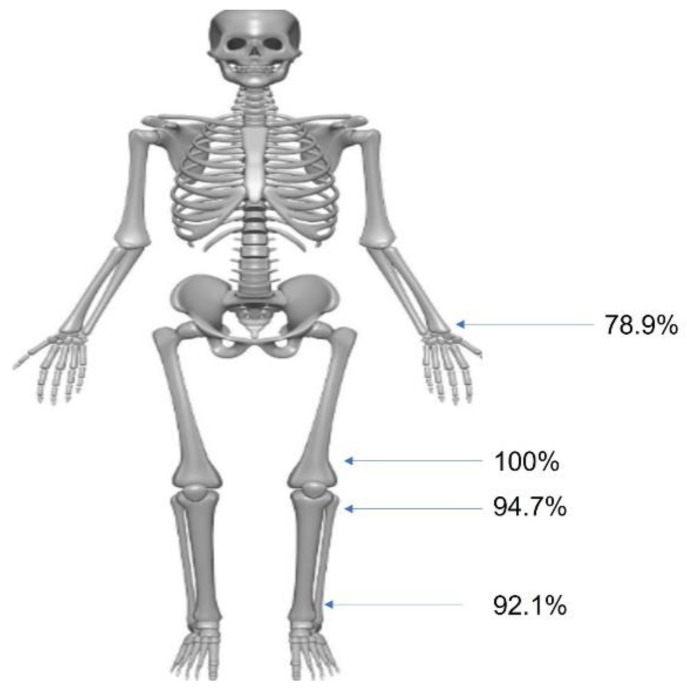
Distribution of involved anatomical sites. The most common site was distal femur and proximal tibia.

The distribution of involved anatomical sites is described in Table 4 and Figure 5.

The mean number of exostoses was 24.3 ± 10.44 (range, 7–52), with the most common sites being the distal femur (100%) and proximal tibia (94.7%). The characteristics of the deformities at each site are described in Table 5. Bilateral involvement and deformities were more common in lower than in upper extremities (27.5% vs. 55.8% and 60.0% vs. 76.74%, respectively). Hand or foot involvement was detected in 14 and 9 patients, respectively. Deformity was more common in the hand than in the foot (50.0% vs. 22.2%). In total, 17 patients showed deformities in all joints of the hip, knee, and ankle. We did not find any factors affecting this upon multivariate logistic regression analysis.

**Figure 6:**
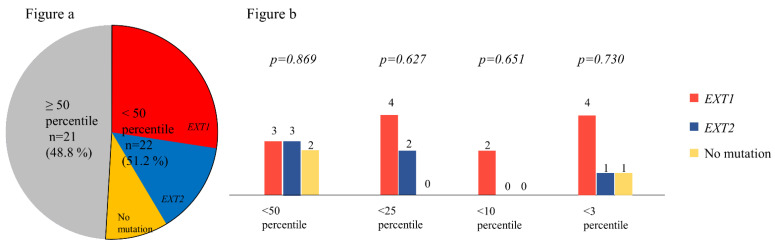
(**a**) Distribution of height percentile according to the gene mutation. The proportions of patients with EXT1 mutations were higher than those with EXT2 or no mutations among patients with below the 50th percentile of height. (**b**) Distribution of patients in different percentiles of height. There was no significant difference according to the gene mutation.

A height evaluation revealed that heights of 22 (51.2%) of the patients were below the 50th percentile. Although the difference was not statistically significant (*p* = 0.669), a higher proportion of patients with EXT1 mutations were below the 50th percentile of height (Figure 6a). The distribution of patients on a percentiles graph for height is shown in Figure 6b. We found no significant difference in the heights of patients with mutations in the studied genes. In addition, there was no significant difference in height distribution of the two sexes.

Missense mutations were detected in 4 of the 38 patients (10.5%) from 2 families (B, O). In 3 of these patients, mutations were detected in EXT1; in 1 patient, they were detected in EXT2. Two patients with missense mutations had moderate clinical severity, while 2 had severe disease.

**Figure 7:**
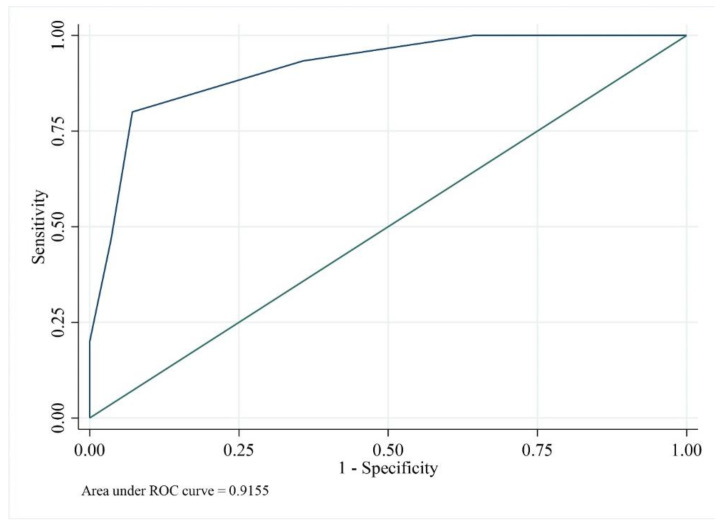
Receiver operating characteristic (ROC) curve used to determine cut-off-values of the deformity score. The deformity score was the only factor which showed association with the ‘severe’ class phenotype (*p* = 0.031) in the multivariate analysis. The deformity score of 1.5 or higher was noted to be threshold for having the ‘severe’ class with 0.9155 of AUC.

We performed a multivariate logistic regression analysis to identify predictors of mild and severe clinical phenotypes (Table 6). The factors that could influence clinical phenotype were the type of mutation, sex, history of familial inheritance, number of exostoses, and deformity score. In univariate analysis, the type of mutation, total number of exostoses, and deformity score were associated with severe disease; however, in the multivariate analysis, the only factor associated with severe disease was the deformity score (*p* = 0.031). ROC curves were used to determine cut-off values of the deformity score (Figure 7). A deformity score of 1.5 or higher was noted to be the threshold for having severe disease, with an AUC of 0.9155.

## 4. Discussion

A limited number of studies regarding genotype-phenotype correlations in patients with MHE have been performed [15,16,17,18,19]. A total of 90.7% of patients in our study had identifiable mutations, which is similar to those in previous studies. These studies employed their own clinical evaluation systems. In 2001, Pedrini et al. classified their patients based on the presence of deformities and functional limitations [18]. Compared to existing classification systems at the time, these criteria were simpler and easier to apply in clinical settings. At the same time as maintaining functional aspects, we have designed a new scoring system (DeNuLF—based on deformity, number of exostoses, LLD, and functional limitations) by adding LLD as a factor. We suggest that patients with MHE who have LLD may experience functional limitations and discomfort. When the LLD > 2.0 cm, they may require surgical treatment.

Our classification system showed that the AUCs were 0.783 and 0.786, respectively, for EXT1 vs. EXT2 and EXT1 vs. no mutation. This finding strengthens the proposed system, which is easier to apply. In our classification system, the score for deformities is 4 points, which is higher than other factors. This score arrangement results in higher AUC than when the scores were assigned in other ways, perhaps because deformity score has a greater influence than other factors. The fact that the deformity score was associated with severe patients supports this analysis and, based on the findings of previous studies, our system includes ‘pain’ as a functional limitation [15,25]. Overall, 17 patients (37.8%) complained of pain and their mean total number of exostoses were 23.9 (range, 7–45). Although we did not observe any relationship between pain and the number of exostoses, pain is a commonly unaddressed symptom in patients with MHE. We found that patients with EXT1 mutations have higher deformity scores, a greater number of exostoses, and a higher total clinical score than those of patients with EXT2 mutations, which are reflected in the severity of our classification system. The severity of our classification system depended on the type of gene mutation (EXT1, EXT2 or no mutation) present (*p* = 0.011); we found that there were no patients without mutations in the ‘severe’ class. This result could be explained by the fact that EXT1 and EXT2 have different roles in heparan sulfate synthesis [6]. EXT1 exerts a predominantly biological function, whereas EXT2 is thought to assist in the folding and transport of EXT1 to the Golgi complex [6]. This is supported by the observation that EXT2 does not have a glycosyltransferase activity if it is not coupled with EXT1 [15]; thus, EXT1 mutations could more severely impair the biological activity of EXT1-EXT2 dimers, leading to a severe clinical phenotype.

Missense mutations were detected in four patients (10.5%) in our cohort, which varies from other studies [1,14,18,21,26]. The missense mutation c.1037 G>T (p.Arg346Ile), which disrupts p.Arg346 amino acid residue, was discovered in our analysis. Other variants that disrupt this residue have been observed in individuals with EXT1 mutations [1,14,27], suggesting that it is a clinically significant residue. Although evidence indicates that the variant is pathogenic [1,14,27], further studies will be necessary to prove this conclusively. We observed the nonsense mutation c.67C>T (p.Arg23X)—which has been reported as a possible hotspot in Italian MHE patients [14,27]—was also recently observed in an Indian patient [21], and the hypothesis of common ancestry may need to be taken into consideration again. An E6 or E6–E8 heterodeletion in EXT2 was found in five patients (27.8%) and three families, which is a large proportion compared to that in the previous study (27.8% vs. 3.8%) [14]; further studies are needed to determine whether this mutation is related to a specific ancestry origin and to find possible hotspots for E6 or E6–E8.

In the multivariate analysis, the deformity score was the only factor associated with severe disease (Table 6). Univariate analysis also linked the number of exostoses and the type of EXT gene with ‘severe class’ disease. Pedrini et al. [18] have reported that the factors associated with severe disease class were being male, having an EXT1 mutation, and having more than 20 sites of exostoses, which is consistent with our findings. Furthermore, it is important to note that in our study, a higher deformity score was associated with more severe disease. Close monitoring for the development of severe disease is required in patients with EXT1 mutations and a higher number of exostoses, as well as in patients with high deformity scores. Of note, deformities are observed more often in the lower extremities than in the upper extremities and in the hand than in the foot (Table 5).

The study has several limitations. First, this was a cross-sectional study, and we did not evaluate age-related factors. Therefore, confounding factors may be involved. Second, the small sample size may have contributed to a degree of bias. The small sample size may also have affected the results of the multivariate regression analysis. 

## 5. Conclusions

In conclusion, 90.7% of patients with MHE showed mutations in EXT1 or EXT2. Our own scoring system, based on the deformity of each segment, number of exostoses, LLD, and functional limitations, showed reliable results with an AUC of 0.78. Our proposed system also included an evaluation of functional impairment. We found that the degree of deformity is an important factor in determining the phenotype of MHE and recommend close monitoring for the development of severe disease in patients with high deformity scores.

## Figures and Tables

**Table 1:** Scoring system ‘De-Nu-L-F’ used in this study.

Parameters	Score
Deformity	Maximum of 4
Upper (except hand/ elbow dislocation = 1)	0.5 (1)
Lower (except foot)	0.5
Hand	0.5
Foot	0.5
Additional	
Both	0.5
Hip and knee and ankle	0.5
Number of masses	Maximum of 2
≥20	
10≤ <20	1
<10	
LLD	Maximum of 2
≥20	2
10≤ <20	1
Function	Maximum of 2
Pain	
Limited ROM	

Mild: A total score of <4; Moderate: a total score 4 ≤ <6; Severe: a total score >6.

**Table 2:** Clinical assessment according to EXT1/EXT2 mutation.

	EXT1 *n* = 21	EXT2 *n* = 18	No Mutation *n* = 4	*p*-Value	EXT1 vs. EXT2	EXT 1 vs. No Mutation	EXT2 vs. No Mutation
Total clinical score Mean (±SD)	5.76 (±1.60)	4.06 (±1.47)	4.63 (±1.44)	0.005 ^1^	0.004	0.544	>1.0
Deformity score Mean (±SD)	1.86 (±0.88)	0.94 (±0.84)	1.13 (±0.48)	0.018 ^1^	0.004	0.367	>1.0
Number of exostoses Mean (±SD), range	29.19 (±8.18), 12–52	20.11 (±8.18), 7–39	21.75 (±9.91), 7–28	0.018 ^1^	0.017	0.499	>1.0
Severity				0.011 ^2^			
Mild	2 (9.5%)	8 (44.4%)	1 (25.0%)		–	–	–
Moderate	7 (33.3%)	7 (38.9%)	3 (75.0%)		–	–	–
Severe	12 (57.1%)	3 (16.7%)	0 (0.0%)		–	–	–
Gender				0.814 ^2^			
Male	9 (42.9%)	10 (55.6%)	2 (50.0%)		–	–	–
Female	12 (57.1%)	8 (44.4%)	2 (50.0%)		–	–	–
Familial or sporadic				0.066 ^2^			
Sporadic	2 (9.5%)	0 (0.0%)	2 (28.6%)		–	–	–
Familial	18 (85.7%)	17 (94.4%)	2 (71.4%)		–	–	–
Unknown	1 (4.8%)	1 (5.6%)	0 (0.0%)		–	–	–
Mutation variant				0.076 ^2^			
Nonsense	1 (4.8%)	4 (22.2%)	–		–	–	–
Missense	4 (19.0%)	2 (11.1%)	–		–	–	–
Frameshift	8 (38.1%)	6 (33.3%)	–		–	–	–
Splice-site	0 (0.0%)	1 (5.6%)	–		–	–	–
Big deletion or duplication							

SD: standard deviation, ^1^: ANOVA, ^2^: Fisher’s exact test.

**Table 3:** Gene mutations in patients.

Patients	Family	Familial History	Sex	Clinical Class (Score)	EXT1	EXT2	Protein Change	Reference	Variant Type
1	A	f	M	Moderate (5.5)		c.1103delA	p.Glu368fs	Present study	Small deletion (Frameshift)
2	B	f	M	Moderate (5.5)	c.1037 G>T		p.Arg346Ile	Jennes et al. [14]	Missense (likely pathogenic)
3	B	f	F	Moderate (5.0)	c.1037 G>T		p.Arg346Ile	Jennes et al. [14]	Missense (likely pathogenic)
4	B	f	M	Severe (7.0)	c.1037 G>T		p.Arg346Ile	Jennes et al. [14]	Missense (likely pathogenic)
5	C	f	F	Severe (6.5)		E6, hetero deletion		Present study	Single exon deletion
6	D	f	F	Moderate (4.0)		E6-E8, hetero deletion		Present study	Multiple exon deletion
7	D	f	M	Mild (2.0)		E6-E8, hetero deletion		Present study	Multiple exon deletion
8	E	f	M	Mild (3.5)		c.610delG	p.Asp204fs	Present study	Small deletion (Frameshift)
9	E	f	M	Moderate (4.0)		c.610delG	p.Asp204fs	Present study	Small deletion (Frameshift)
10	F	f	F	Mild (3.0)	Whole gene, hetero deletion			Present study	EXT1 All Exon deletion
11	F	f	F	Severe (6.5)	Whole gene, hetero deletion			Present study	EXT1 All Exon deletion
12	F	f	F	Severe (8.0)	Whole gene, hetero deletion			Present study	EXT1 All Exon deletion
13	G	f	M	Severe (6.5)	c.112G>T		p.Glu38Ter	Present study	Nonsense
14	H	f	F	Mild (3.0)		c.699T>G	p.Tyr233Ter	Wuyts et al. [20]	Nonsense
15	H	f	M	Moderate (5.5)		c.699T>G	p.Tyr233Ter	Wuyts et al. [20]	Nonsense
16	I	f	M	Mild (3.0)		c.67C>T,	p.Arg23Ter	Malini et al. [21]	Nonsense
17	I	f	F	Moderate (5.0)		c.67C>T	p.Arg23Ter	Malini et al. [21]	Nonsense
18	J	f	F	Mild (2.5)	No mutation	No mutation	–		–
19	K	f	M	Moderate (5.0)	No mutation	No mutation	–		–
20	L	s	F	Severe (6.5)	c.453delG		p.Ala151fs	Present study	Small deletion (Frameshift)
21	M	f	M	Severe (6.0)		c.779dup	p.Gly259fs	Present study	Small duplication (Frameshift)
22	N	f	M	Moderate (4.5)	E1-E2, triplicated			Present study	Exon Triplication
23	O	f	M	Severe (6.0)	c.1019G>T		p.Arg340Leu	Hecht et al. [22]	Missense (pathogenic)
24	P	f	F	Moderate (4.0)	E1 heterodeletion			Present study	E1 heterodeletion
25	P	f	F	Severe (7.0)	E1 heterodeletion			Present study	E1 heterodeletion
26	P	f	F	Mild (2.0)	E1 heterodeletion			Present study	E1 heterodeletion
27	P	f	F	Severe (7.0)	E1 heterodeletion			Present study	E1 heterodeletion
28	R	f	F	Moderate (4.5)		E6-E8, heterodeletion		Present study	Multiple exon deletion
29	S	f	F	Moderate (4.5)		c.1103delA	p.Glu368fs	Present study	Small deletion (Frameshift)
30	T	u	F	Moderate (4.0)	c.1469delT		p.Leu490fs	Ahn et al. [7]	Small deletion (Frameshift)9
31	U	s	M	Moderate (5.5)	No mutation	No mutation	–		–
32	V	s	F	Moderate (5.5)	No mutation	No mutation	–		–
33	W	f	F	Mild (2.0)	c.1345-1366del	c.484C>T	p.Gln162Ter	Nykamp et al. [23]	Nonsense
34	X	f	M	Moderate (5.0)			p.Pro449fs	Present study	Small deletion (frameshift)
35	Y	f	M	Severe (6.0)	c.536dupA		p.Gln179fs	Present study	Small duplication (Frameshift)
36	Y	f	F	Severe (6.5)	c.536dupA		p.Gln179fs	Present study	Small duplication (Frameshift)
37	Y	f	M	Severe (7.5)	c.536dupA		p.Gln179fs	Present study	Small duplication (Frameshift)
38	Y	u	M	Severe (6.0)		c.1080-2del			Splice site
39	Z	s	M	Severe (8.0)	c.247dupC		p.Arg83fs		Small duplication (Frameshift)
40	A2	f	M	Mild (2.5)		c.1103delA	pGlu368fs	Present study	Small deletion (Frameshift)
41	B2	f	F	Moderate (5.5)	c.89_95delCATCGAG		p.Ala30fs	Present study	Small deletion (Frameshift)
42	C2	f	F	Mild (2.0)		c.514C>T	p.Gln172Ter	Richards et al. [24]	Nonsense
43	D2	f	M	Mild (3.5)		E6-E8, heterodeletion	–	Present study	E2 heterodeletion

Reference sequences for EXT1: NM_000127.2. and EXT2: NM_207122.1, f: familial history (+), s: sporadic, u: unknown familial history. f: Family history (+), s: sporadic, u: unknown.

**Table 4:** Distribution of involved anatomical sites.

Sites	*n* (%)
Proximal humerus	6 (15.8%)
Shaft of humerus	26 (68.4%)
Distal humerus	4 (10.5%)
Proximal Ulna	9 (23.7%)
Distal Ulna	30 (78.9%)
Proximal Radius	11 (28.9%)
Distal Radius	26 (68.4%)
Hand	11 (28.9%)
Ilium	17 (44.7%)
Ischium	12 (31.6%)
Proximal Femur	35 (92.1%)
Distal Femur	38 (100%)
Proximal Tibia	36 (94.7%)
Distal Tibia	35 (92.1%)
Proximal Fibula	34 (89.5%)
Distal Fibula	16 (42.1%)
Foot	8 (21.1%)

**Table 5:** Characteristics of deformity in patients.

	Upper Extremity	Lower Extremity	Short Bones	
			Hand	Foot
Involvement (a)	40	43	14	9
Bilateral involvement (b)	11	24	-	-
Proportion of both side involvement (b/a × 100)	27.5%	55.8%	-	-
Deformity (c)	24	33	7	2
Proportion of deformity (c/a × 100)	60.0%	76.74%	50.0%	22.22%

**Table 6:** Factors associated with the clinical class of ‘severe’ using multivariate regression model.

Factors		Univariate				Multivariate			
		Odds Ratio	95% CI		*p*-Value	Odds Ratio	95% CI		*p*-Value
Gene	EXT1	Reference	-	-	-				
	No	N/A	N/A	N/A	N/A				
	EXT2	0.150	0.033	0.680	0.014	1.745	0.181	16.842	0.630
Sex	Male	Reference	-	-	-				
	Female	0.758	0.216	2.666	0.666				
Familial history	Familial	0.480	0.028	8.348	0.614				
	Sporadic	1.000	0.034	29.807	1.000				
	Unknown	Reference	-	-	-				
Total number of exostoses		1.206	1.071	1.358	0.002	1.084	0.943	1.246	0.259
Deformity score		23.984	3.388	169.784	0.001	9.864	1.230	79.132	0.031

N/A: not applicable, CI: confidence interval.

## Data Availability

Deidentified individual participant data (including data dictionaries) will be made available, in addition to study protocols, the statistical analysis plan, and the informed consent form. The data will be made available upon publication to researchers who provide a methodologically sound proposal for use in achieving the goals of the approved proposal. Proposals should be submitted to stjung@jnu.ac.kr.
